# Estrogen receptor beta impacts hormone-induced alternative mRNA splicing in breast cancer cells

**DOI:** 10.1186/s12864-015-1541-1

**Published:** 2015-05-09

**Authors:** Dougba Noel Dago, Claudio Scafoglio, Antonio Rinaldi, Domenico Memoli, Giorgio Giurato, Giovanni Nassa, Maria Ravo, Francesca Rizzo, Roberta Tarallo, Alessandro Weisz

**Affiliations:** Laboratory of Molecular Medicine and Genomics, Department of Medicine and Surgery, University of Salerno, Via S. Allende, 1, Baronissi, SA 84081 Italy; UFR Sciences Biologiques, Université Peleforo Gon Coulibaly, Korhogo, Ivory Coast; Department of Molecular and Medical Pharmacology, University of California, Los Angeles, USA; Molecular Pathology and Medical Genomics, “SS. Giovanni di Dio e Ruggi d’Aragona - Schola Medica Salernitana” Hospital of the University of Salerno, Salerno, Italy

**Keywords:** Breast cancer, Estrogen receptor beta, Alternative splicing, Alternative promoters, RNAseq

## Abstract

**Background:**

Estrogens play an important role in breast cancer (BC) development and progression; when the two isoforms of the estrogen receptor (ERα and ERβ) are co-expressed each of them mediate specific effects of these hormones in BC cells. ERβ has been suggested to exert an antagonist role toward the oncogenic activities of ERα, and for this reason it is considered an oncosuppressor. As clinical evidence regarding a prognostic role for this receptor subtype in hormone-responsive BC is still limited and conflicting, more knowledge is required on the biological functions of ERβ in cancer cells. We have previously described the ERβ and ERα interactomes from BC cells, identifying specific and distinct patterns of protein interactions for the two receptors. In particular, we identified factors involved in mRNA splicing and maturation as important components of both ERα and ERβ pathways. Guided by these findings, here we performed RNA sequencing to investigate in depth the differences in the early transcriptional events and RNA splicing patterns induced by estradiol in cells expressing ERα alone or ERα and ERβ.

**Results:**

Exon skipping was the most abundant splicing event in the post-transcriptional regulation by estradiol. We identified several splicing events induced by ERα alone and by ERα + ERβ, demonstrating for the first time that ERβ significantly affects estrogen-induced splicing in BC cells, as revealed by modification of a subset of ERα-dependent splicing by ERβ, as well as by the presence of splicing isoforms only in ERβ + cells. In particular, we observed that ERβ + BC cell lines exhibited around 2-fold more splicing events than the ERβ- cells. Interestingly, we identified putative direct targets of ERβ-mediated alternative splicing by correlating the genomic locations of ERβ and ERα binding sites with estradiol-induced differential splicing in the corresponding genes.

**Conclusions:**

Taken together, these results demonstrate that ERβ significantly affects estrogen-induced early transcription and mRNA splicing in hormone-responsive BC cells, providing novel information on the biological role of ERβ in these tumors.

**Electronic supplementary material:**

The online version of this article (doi:10.1186/s12864-015-1541-1) contains supplementary material, which is available to authorized users.

## Background

Breast cancer (BC) is the most frequent cancer in women worldwide [1], and its development and progression are known to rely strongly on the stimulation by female sexual hormones, especially estrogens. Estrogen receptors α (ERα) and β (ERβ) are transcription factors that mediate the actions of estrogens in target cells [2,3]. Ligand binding to ERα or ERβ induces receptor dimerization, either as homodimers (ERα/ERα or ERβ/ERβ) or heterodimers (ERα/ERβ) [4], and promotes its translocation to the nucleus and binding of target chromatin sites via Estrogen Response Elements (EREs) and other regulatory elements on DNA [5]. Although encoded by different genes, ERα and ERβ share the same general modular protein structure of the nuclear hormone receptor superfamily, and have almost 100% amino acid sequence homology in their DNA-binding domain. They also show 59% amino acid homology in their ligand-binding domains [4,5]. The two ERs are thus quite similar in sequence and structure, but ERβ has considerably different and, in most cases, opposite biological effects compared to ERα in BC cells, both *in vivo* and *in vitro*. ERα regulates the transcription of hundreds of genes [6], enhancing BC cell growth, proliferation and survival in response to estrogens [7]. The specific role of ERβ and its impact in BC are unclear. This ER subtype is expressed in 70% of human breast tumors in combination with ERα, even if some human breast tumors express only ERβ [8-10]. Several reports have suggested that ERβ has anti-proliferative action in BC cells, by increasing the expression of anti-proliferative genes and/or decreasing the expression of proliferative and anti-apoptotic genes [11-15] and the ERα/ERβ ratio determines the cell-specific response to estrogen. In BC this ratio is higher than in normal tissues, due to up-regulation of ERα and down-regulation of ERβ [16]. Loss of ERβ mRNA levels in cancer can occur as a result of promoter methylation [17]. These observations have suggested a positive prognostic value of this receptor subtype [12,18]. However, several studies have reported also a negative prognostic value for ERβ expression [19,20], making the overall contribution of this receptor isoform to BC biology unclear.

The exact mechanism of the antagonism between ERβ and ERα is only partially known. As the two receptors share only 30% homology in their transactivation domain AF1 [5], it is likely that they show different patterns of interaction with coregulatory proteins. Indeed, we have previously reported that the protein interactomes of both ERβ and ERα show significant differences of the protein complexes engaged by the two ER subtypes [21-25]. A particularly interesting subset of interacting proteins, with only partially overlapping interaction patterns between the two receptors [26], comprises factors involved in RNA maturation and splicing [Additional file 1: Figure S1] [26].

Alternative splicing is a mechanism by which cells can increase the variability of their proteomes by changing the composition of transcribed genes through differential choice of exons to be included in the final mRNA molecule [27]. Almost 90% of human genes show alternative splicing during development, cell differentiation and disease [28]. Recent studies have shown the existence of cancer-specific splicing events by which transformed cells switch from the adult isoform of the gene to a more embryonic one, contributing to the cancer phenotype [29-31].

Alternative splicing events have been monitored in BC and in numerous tumor types [32], and ERα itself has been reported to induce alternative splicing of a specific set of genes [33-35]. Here, we investigated the ability of ERβ to regulate mRNA maturation and splicing in hormone-responsive BC cells. To this purpose, we performed high-throughput RNA sequencing (RNA-seq) analysis of human MCF-7 cell lines stably transfected with ERβ, and compared them with the wild type line, expressing only ERα, upon 17β-estradiol (E2) stimulation.

## Results

### High-throughput sequencing in ERβ + and ERβ- human BC cell lines

We have previously established and characterized subclones of the human BC cell line MCF-7 expressing human ERβ fused to a Tandem Affinity Purification (TAP) tag, and have shown that the addition of the TAP-tag at either the N- or the C-terminus of the protein (indicated as Nt-ERβ and Ct-ERβ, respectively) does not alter significantly the receptor function, nor its ability to activate transcription or to antagonize ERα-dependent transcription [21,22,36]. In order to study the early events of hormone-induced pre-mRNA maturation, we used these stable cell lines to perform deep-sequencing analysis of estrogen-induced transcriptional events shortly after stimulation with 17β-estradiol (2 h), to focus mostly on primary transcriptional events [21,36]. For comparison, we also performed the experiment in wild-type MCF-7 cells, which do not express endogenous ERβ.

**Figure 1:**
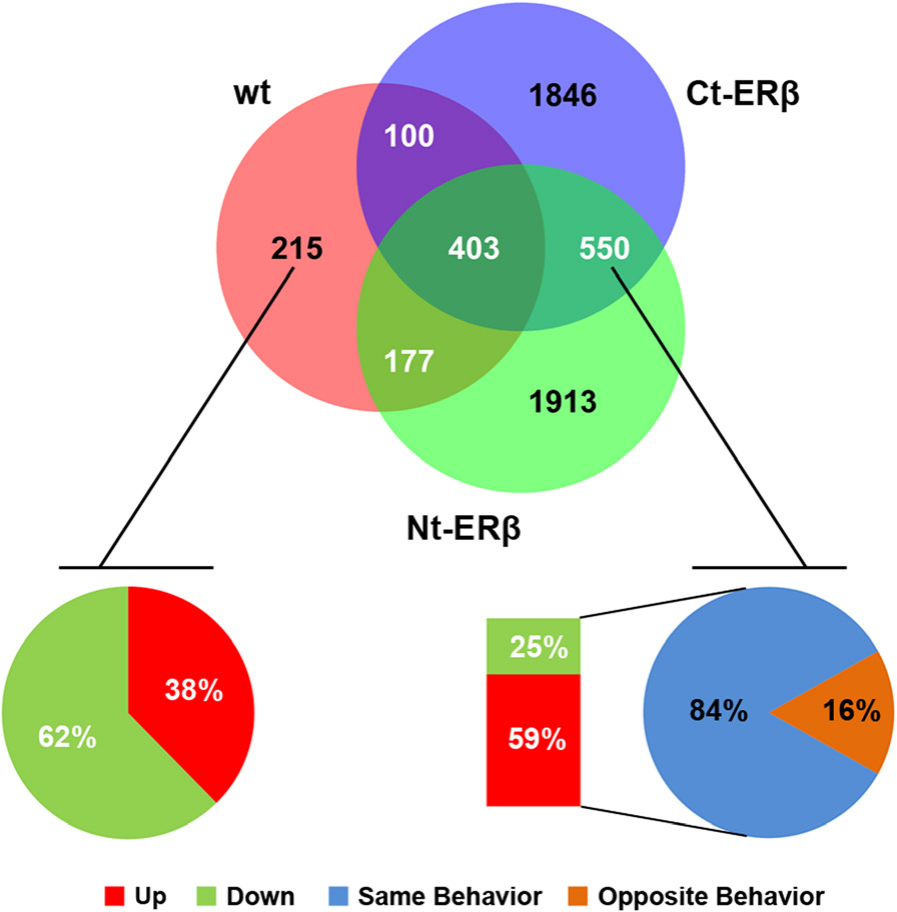
Venn diagram of expressed genes in ERβ + and wt breast cancer cell lines. The Venn diagram shows the number of genes regulated by estradiol in the three cell lines, as indicated: wt (parental MCF-7 cells); Ct-ERβ (MCF-7 subclone stably expressing ERβ tagged with the TAP-tag at the C-terminus); Nt-ERβ (MCF-7 subclone stably expressing ERβ tagged with the TAP-tag at the N-terminus). The pie charts in the lower panels specify the direction of regulation by estradiol (induction or repression) of the genes whose regulation is present in wt cells but is lost in both ERβ + cell lines (left panel), or of the genes whose regulation by estradiol is not present in the wt cells but appears in both ERβ + lines (right panel).

Almost 70 million reads/replicate were aligned against the reference human genome for ERβ- and ERβ + BC cell lines. The number of reads for genes and isoforms were normalized to “Fragment Per Kilobases of exon per Million of mapped reads” (FPKM). In order to analyze genes and isoforms, we set 0.5 FPKM (at least one analyzed condition, with/without E2 stimulus) as the expression level threshold. In this way, we identified 16,821 (MCF-7 wt), 16,148 (Ct-ERβ) and 17,135 (Nt-ERβ) genes as expressed. The criteria for considering genes and isoforms as significantly regulated by estradiol were: FPKM value ≥0.5 in at least one analyzed condition, *q*-value (FDR-adjusted *p*-value of the test statistics) ≤0.05 and |fold-change| (FC) ≥1.3 [Additional file 2: Table S1]. As shown in Figure 1, 895 (MCF-7 wt), 2,899 (Ct-ERβ) and 3,043 (Nt-ERβ) genes were detected as significantly regulated by E2 in these BC cell lines. Expression of ERβ in MCF7 cells significantly affected the estrogen-dependent gene expression profile: the regulation of around 230 genes (≈25% of E2-regulated genes) was lost in both cell lines expressing ERβ, while a large number of genes which were not regulated in *wt* cells became significantly regulated in Ct-ERβ (2,396) and Nt-ERβ (2,463) clones (Figure 1). The genes regulated consistently in both ERβ + lines are reported in Additional file 2: Tables S1-D. Interestingly, expression of ERβ in this BC cell line had a stronger effect on inhibited genes than on the activated ones: regulation of 40% of genes inhibited by estradiol in *wt* cells was lost in both ERβ + cell lines, versus 14% for estrogen-activated genes. Gene Ontology analysis revealed that among the most enriched functions in the group of genes whose regulation by estradiol was lost in both ERβ + cells there were, as expected, DNA Replication, Recombination and Repair, as well as Cell Cycle and Cell Morphology (data not shown).

### Estrogen-dependent splicing events in ERβ + and ERβ- human BC cell lines

**Figure 2:**
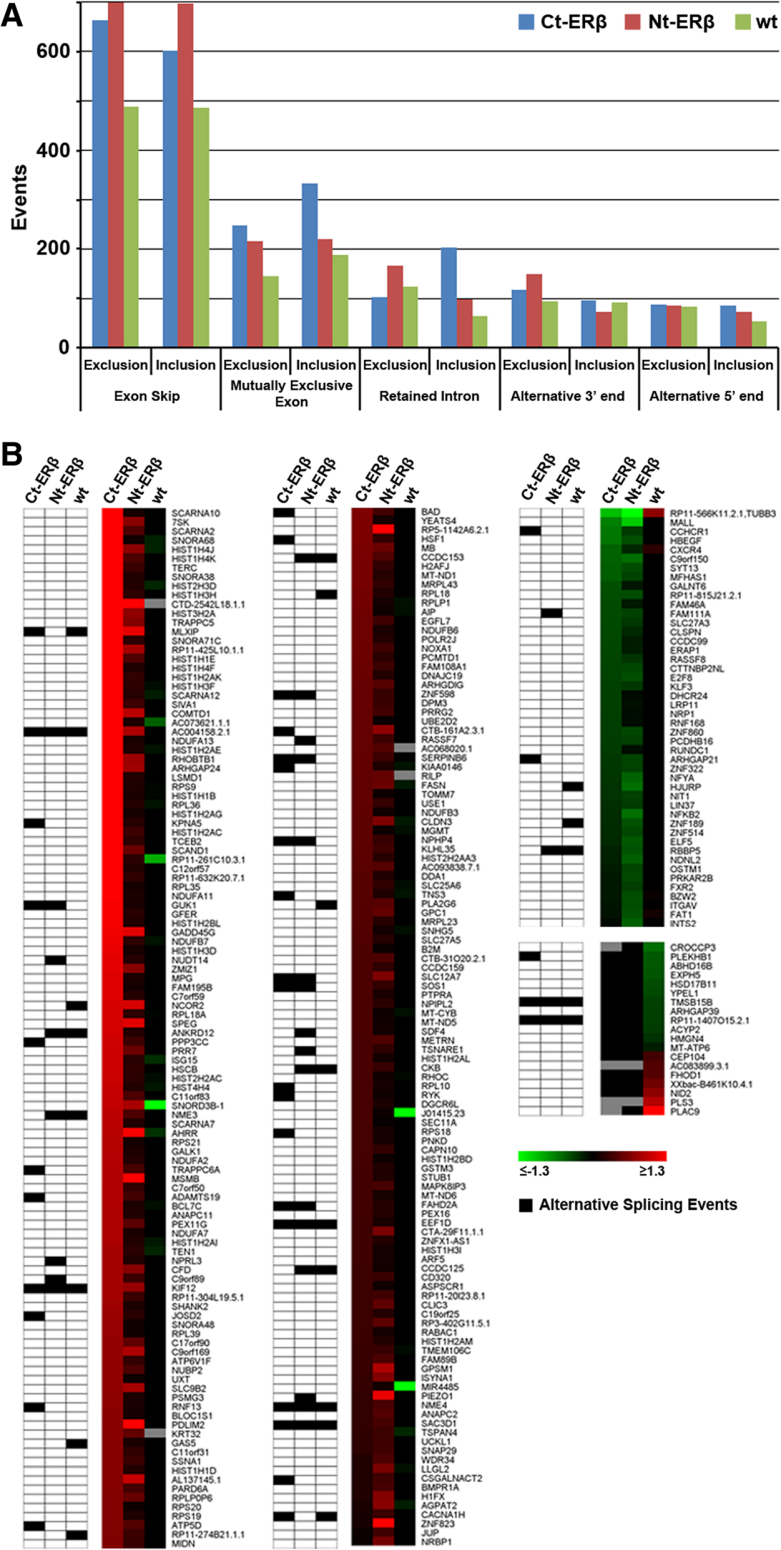
Annotation of splice events in ERα + and ERα + ERβ + BC cell lines. **(A)** The bar plot shows the number of all alternative splicing events occurring in the cell lines analyzed. Inclusion and exclusion behavior for each event are shown (FDR ≤ 0.05; c ≤ |0.1|). **(B)** Genes whose regulation has opposite direction in the ERβ + lines compared to the wt MCF-7. The heat map on the right side shows the gene expression fold changes induced by estradiol. The matrix on the left side shows in black those genes for which a splicing event was detected in at least one of the cell lines. The nomenclature for the cell lines is the following: wt (parental MCF-7 cells); Ct-ERβ (MCF-7 subclone stably expressing ERβ tagged with the TAP-tag at the C-terminus); Nt-ERβ (MCF-7 subclone stably expressing ERβ tagged with the TAP-tag at the N-terminus).

Events of exon skipping, mutually exclusive exons, alternative start, stop splice site and intron retention were annotated using the Multivariate Analysis of Transcript Splicing (MATS) software [37] [Additional file 3: Tables S2]. Exon skipping appeared to be the predominant splice event in all cell lines analyzed. MATS reveled in detail 1,264 (Ct-ERβ), 1,402 (Nt-ERβ) and 975 (MCF-7 wt) exon skipping events induced by estradiol, associated with 1,016 (Ct-ERβ), 1,117 (Nt-ERβ) and 816 (*wt* MCF-7) genes (Figure 2A). Five hundred seventy-five events were common to all the cell lines analyzed while 115 showed opposite exon inclusion level in ERβ + lines compared to ERβ- *wt* cells. We also observed high levels of retained intron and mutually exclusive exons events, confirming a complex and significant effect of ERs on the regulation of RNA splicing in these cell lines (Figure 2A).

To focus on the differences in splicing patterns between ERβ + and ERβ- cell lines, we first looked at the genes which were regulated by estradiol in opposite direction in ERβ + cells versus *wt* cells [Additional file 4: Table S3]. Among 298 regulated genes, 56 also underwent estradiol-induced alternative splicing in at least one of the cell lines, confirming that pre-mRNA maturation was regulated concurrently with transcription in a significant fraction of ERβ-regulated genes (Figure 2B). These ERβ-regulated genes undergoing alternative splicing included transcriptional regulators (*NCOR2*, *ZNF189*, *MLXIP*, *ANKRD12*, *HSF1*), enzymes involved in nucleoside/nucleotide metabolism (*GUK1*, *NME3*, *NME4*), actin remodeling and cellular transport processes (*TNS3*, *TRAPPC6A*, *TMSB15B*, *KIF12*), and protein translation (*ZNF98*, *EEF1D*, *RPL10*, *RPL18*, *RPS18*).

**Figure 3:**
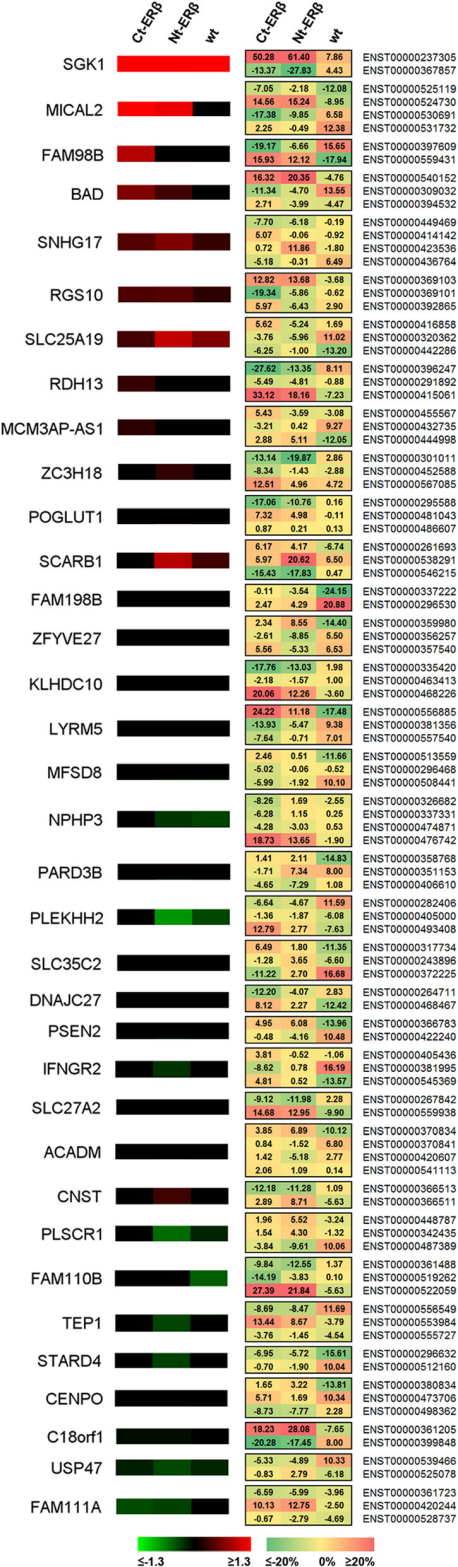
Selected isoform switches affected by the expression of ERβ. The ERβ-dependent differential splicing events that affect most prominently the balance of different isoforms for each gene were identified by using the following parameters: (i) isoform ratio (FPKM_isoform_/FPKM_gene_ %) changing of at least 10% after estradiol stimulation, either in the wt cells or in both the ERβ + cells in at least one of the isoforms of the gene; (ii) at least one isoform of the gene significantly regulated by estradiol (*p*-value ≤ 0.05; |FC| ≥ 1.3) either in the wt cells or in both the ERβ + cells; (iii) isoform ratio changing in opposite direction in the wt cells compared to the ERβ + cells; (iv) at least one splicing event identified by MATS analysis. Thirty-five genes satisfied all the selection requirements; of these, only 2 to 3 isoforms were selected for presentation, according to the expression levels and the regulation by estradiol. Left panel: heat map of regulation of the selected genes by estradiol in: Ct-ERβ (MCF-7 subclone stably expressing ERβ tagged with the TAP-tag at the C-terminus); Nt-ERβ (MCF-7 subclone stably expressing ERβ tagged with the TAP-tag at the N-terminus); wt (parental MCF-7 cells). Right panel: heat map with the change in isoform ratio (FPKM_isoform_/FPKM_gene_ %) between E2-treated and non-treated cells in the indicated cell lines; for each gene, at least two different isoforms are presented, to show estrogen-induced switch from one isoform to the other.

**Figure 4:**
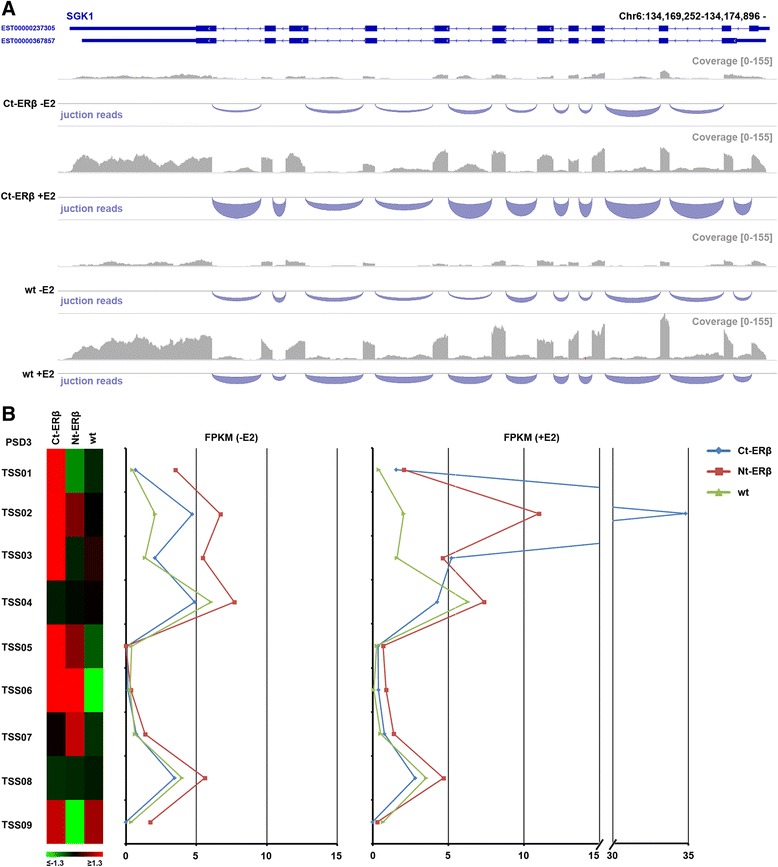
Examples of ERβ-specific splicing events. **A)** Example of alternative splicing in the SGK1 gene. The upper panel shows a schematic representation of two differentially regulated isoforms of the gene (same as those shown in Figure 3 for the same gene), differing in the transcription start site, in the inclusion of the first intron (the gene is encoded by the reverse strand) and in the transcription stop site. The lower panels show a representation of the RNA-Seq reads and junction reads associated with the gene in the different conditions: Ct-ERβ (MCF-7 subclone stably expressing ERβ tagged with the TAP-tag at the C-terminus) without or with E2 stimulation, and wt (parental MCF-7 line) without or with E2 stimulation. **B)** Example of ERβ-specific alternative promoter usage in the gene *PSD3*. The left panel shows a heat map with the fold change of all the primary transcripts associated with the gene in: Ct-ERβ (MCF-7 subclone stably expressing ERβ tagged with the TAP-tag at the C-terminus); Nt-ERβ (MCF-7 subclone stably expressing ERβ tagged with the TAP-tag at the N-terminus); wt (parental MCF-7 cells). The right panels show the logarithm base 10 (log_10_) FPKM for each of the different transcript start sites (from TSS01 to TSS09) corresponding with the different gene isoforms in the above described cell lines, without (left) or with (right) E2 stimulation.

Estrogen-induced differential splicing has been reported also in genes independently on transcriptional regulation [35]. In order to find the splicing events differentially regulated by estradiol in ERβ + compared to *wt* cells, we scanned the whole list of expressed genes for splicing patterns occurring differentially in ERβ + *vs* ERβ- cells. In addition, we focused on those splicing events whose occurrence significantly altered the ratio between different isoforms of the same gene. Therefore, for each isoform we calculated the percentage of gene expression associated with that particular isoform (FPKM ratio: FPKM_isoform_/FPKM_gene_ %) and selected for further analysis only those isoforms in which estradiol induced a change in FPKM ratio of at least 10% either in *wt* MCF-7 or in both ERβ + cell lines. Other criteria of inclusion were: occurrence of at least one splicing event as detected by MATS; at least one isoform of the gene with FPKM value ≥0.5 in at least one analyzed condition, *q*-value ≤0.05 either in *wt* or in both ERβ + cell lines; regulation in both ERβ + lines in opposite directions compared to the *wt*. In this way, we identified the quantitatively most relevant splicing events differentially regulated by E2 in ERβ + versus ERβ- cells, including 35 genes whose isoform composition changed significantly after E2 stimulation in an ERβ-dependent fashion (Figure 3). Among these, we found genes involved in apoptosis (BAD), lipid metabolism (*ACADM*, *PLSCR1*, *SLC27A2*, *STARD4*), nutrient transport (*SLC25A19*, *SLC35C2*), transmembrane receptor signaling (*IFNGR2*, *LDLRAD4*), Notch signaling (*PSEN2*, *POGLUT1*, *SGK1*, *SLC35C2*), as well as some non-coding RNAs (*MCM3AP-AS1*, *SNHG17*). An example of a gene whose splicing pattern was affected by estradiol in an ERβ-dependent fashion is reported in Figure 4A, showing the gene SGK1, which encodes a serum and glucocorticoid-induced serine/threonine protein kinase involved in ion transport affecting many cellular processes such as cell growth, proliferation, survival, apoptosis and migration [38,39]. The gene was induced by estradiol in both *wt* and ERβ + cells; however, expression of ERβ in the absence of hormonal stimulation induced a promoter usage switch and retention of the first intron causing an alternative translation start site and therefore the expression of an isoform with a different N-terminal sequence (ENST00000367857), compared to the major isoform expressed (ENST00000237305). It has been reported that alternative isoforms at the N-terminus can affect SGK1 localization and protein stability: the ERβ-specific form with intron-retention misses a very crucial sequence involved in targeting to the endoplasmic reticulum as well as in proteasomal degradation [40].

This data suggests that expression of ERβ causes switches in estradiol-induced splicing patterns, potentially affecting expression or function or ER targets.

### Estrogen-dependent alternative promoter usage in ERβ + *vs* ERβ- BC cells

As the usage of alternative promoters is a major determinant of protein diversity, even more than alternative splicing [41,42], we next focused on utilization of multiple promoters. We grouped the primary transcripts of a gene based on the promoter used, and subsequently tested changes in primary transcript abundance by measuring the square root of the Jensen-Shannon divergence that occurred within and between the analyzed groups. Finally, we investigated the potential promoter switch regulation for the genes comprising more than one differentially expressed transcript initiating from distinct genomic loci. There were 977 (Ct-ERβ), 402 (Nt-ERβ) and 222 (*wt* MCF-7) distinct promoter-switching genes (FDR ≤ 0.05) in ERβ + and ERβ- BC cell lines, respectively [Additional file 5: Tables S4A-C]. Of the 222 promoter-switching genes recorded in *wt* MCF-7 cells, 165 did not show promoter switch in both ERβ + cell lines, while 61 new promoter-switching events, not present in *wt* cells, were detected in both ERβ + lines. These 61 ERβ-specific promoter-switching genes are involved in important cellular functions known to be controlled by E2 in BC cells, such as transcription (*FOXJ3*, *GTF2H*, *NR2C2AP*), DNA metabolism and repair (*PRKDC*, *REV3L*, *SCAND3*), pre-mRNA maturation and splicing (*PPM1G*, *PRPF38B*, *RNMT*, *RPRD1A*, *SMG1*), translation (*FARSB*, *RARS*, *RPS21*, *UTP20*), protein ubiquitination and proteasome pathway (*CUL5*, *KLHL2*, *PSMB1*, *USP7*), cytoskeleton and cytokinesis (*DCTN4*, *MYL12A*, *SEPT9*, *SYDE2*), membrane metabolism, remodeling and intracellular transport (*ATP9A*, *CAST*, *MAL2*, *PSD3*, *TMEM43*, *TRAPPC9*), cell adhesion and polarity (*ARHGAP12*, *CD9*, *CLDN7*, *EPB41L5*, *PERP*), signal transduction (*MAP3K5*, *NGFRAP1*, *TNFRSF12A*, *WWC3*, *ZDHHC5*).

As an example, we focused on the *PSD3* gene, predicted to be a nucleotide exchange factor for ADP Ribosylation Factor (ARF) 6, a member of the RAS family involved in vesicular trafficking, remodeling of membrane lipids, and signal transduction [43]. As show in Figure 4B, for this gene we found 9 distinct primary transcripts (TSS01-TSS09) whose usage ratios changed with E2 stimulus (right panel) compared to the control untreated cells (left panel). In ERβ + cells, estradiol induced a switch from promoter TSS08 (ENST00000523619) to the downstream promoter TSS02, resulting in a shorter transcript (ENST00000519653), which is predicted to undergo nonsense-mediated decay [Additional file 6: Table S5].

### Estrogen-dependent splice ratios in ERβ + vs ERβ- cells

To obtain a comprehensive view of the estrogen-induced differences in the splice ratios between *wt*, Ct-ERβ and Nt-ERβ cells, we employed Cuffdiff v2.1.1 [44], which calculates the changes in splice isoforms abundance, by quantifying the square root of the Jensen-Shannon divergence, considering each primary transcripts able to produce multiple isoforms. We determined 217 (Ct-ERβ), 241 (Nt-ERβ) and 95 (MCF-7 wt) differentially spliced genes (DSGs, i.e. genes for which estradiol challenge induced at least one splicing event) with a FDR value <0.05 [Additional file 5: Tables S4D-F]. Of the 95 spliced genes detected in *wt* cells, sixty-nine lacked splicing events in both ERβ + cell lines, suggesting inhibition of ERα-dependent splicing by ERβ. Moreover, 28 ERβ-specific DSGs, in which no estrogen-induced splicing was recorded in *wt* cells, were found in both ERβ + cell lines. These include genes involved in mitosis and cytokinesis (*SEPT9*, *BICD2*, *ENSA*, *PDS5A*), cell cycle control (*CCNJ*, *RAN*), transcription (*C11orf30*, *EIF3M*, *HIPK1*, *PBX1*, *ZNF124*, *ZNF131*), protein folding (*DNAJB6*, *HSP90B1*), ubiquitination and sumoylation (*DCUN1D4*, *SENP5*, *TRIM33*), and signal transduction (*APBB2*, *RTKN2*).

### Correlation between ER binding and ER-dependent splicing

**Figure 5:**
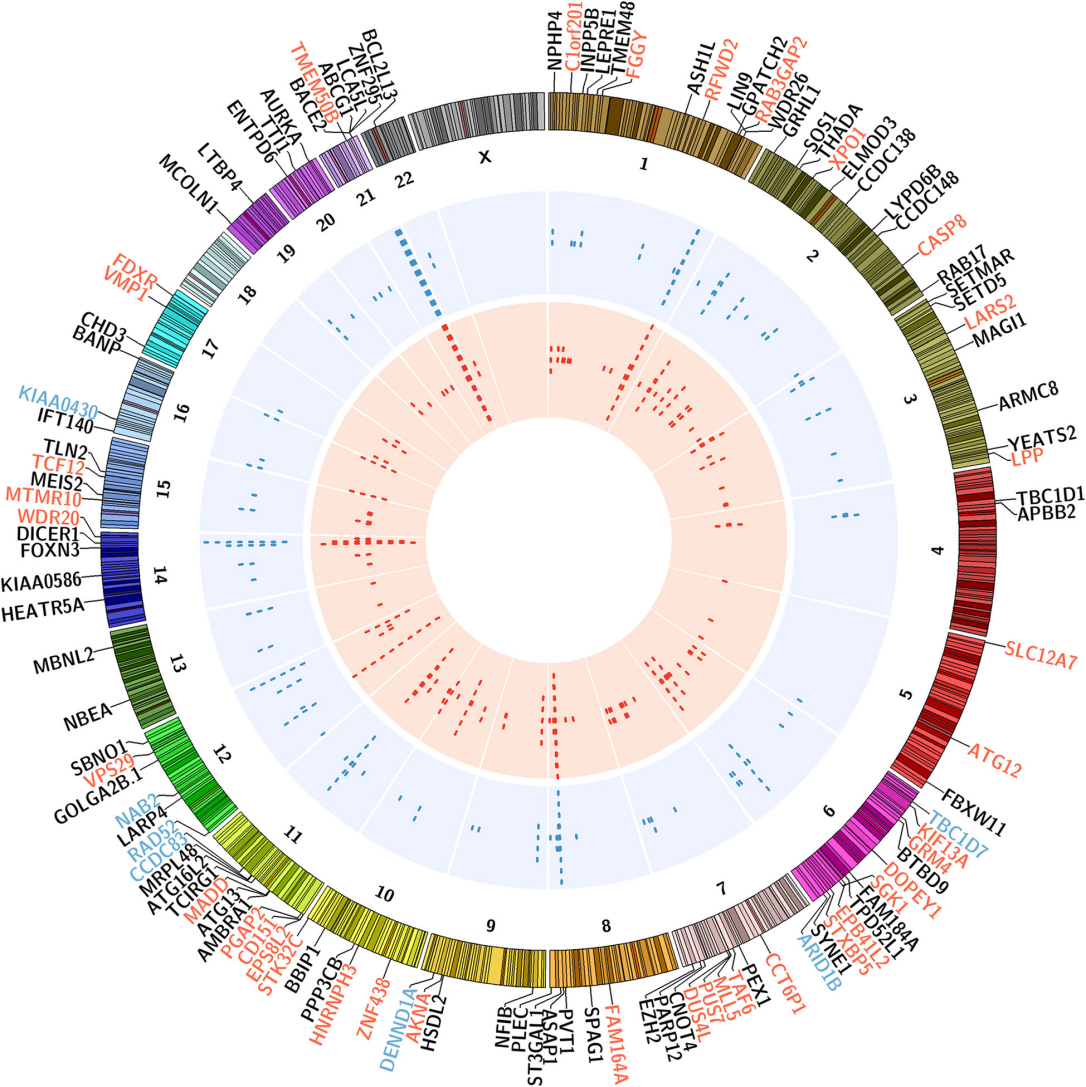
Correlation between ERβ-specific splicing and ER binding. Circos plot of differential spliced genes (DSGs) common to both ERβ + cell lines which contain at least one ER binding event within a window of 10 kB around the gene. The outer ring shows chromosome ideograms with the relative genes located in their respective chromosomal locations, with the following color code: the genes that have both ERβ and ERα binding are in black; the genes which have only ERβ binding are in red; the genes which have only ERα binding are in blue. The two internal rings represent ERα and ERβ binding events in blue and red, respectively.

In order to identify direct splicing targets of ERα and ERβ, we next investigated the presence of ERα and ERβ binding sites in genomic locations close to the identified DSGs [Additional file 7: Table S6]. To verify the specificity of ER binding sites, we created a heat-map based on previously published ChIP-Seq data [21,45]. The binding densities of ERα and ERβ were clustered according to the seqMINER platform [46]. In the clustering shown in [Additional file 8: Figure S2], each line represents a genomic location of a binding site with its surrounding ±1.5 kb region. In the left panel, ERα binding sites were used as a reference to collect a ChIP-seq tag densities window in ERβ + and ERβ- cell lines, while in the right panel ERβ binding sites were used as a reference. The heat map, representing the clustered density matrix, confirmed that ERβ presence modified a significant number of ERα binding sites. In order to investigate the correlation between ERα and ERβ DNA binding and splicing events, we compared our RNA-Seq data with the ChIP-Seq data we had previously obtained in these same cell lines [21]. We considered the binding sites included within regions spanning 10 kb upstream or downstream of all DSGs in each cell line. The Circos plot [47] in Figure 5 shows the DSGs for which estradiol challenge induced at least one splicing event in both ERβ + cell lines with a nearby binding site for ERα and/or ERβ. The outer ring (blue) reports the ERα binding sites, while the inner ring (red) shows the ERβ binding sites. Based on ERα and ERβ binding, we distinguished three different DSGs groups. Group 1 DSGs were associated with both ERα and ERβ binding sites (black). Group 2 includes DSGs associated with ERα binding sites exclusively (blue) and group 3 DSGs associated with ERβ binding sites exclusively (red). The vast majority of genes exhibited both binding sites (Group 1), confirming the competing role of ERα and ERβ. Interestingly, among the putative direct ERβ splicing targets we found many genes involved in transcription: transcription factors (*FOXN3*, *NFIB*, *TAF6*, *TCF12*, *ZNF295*, *ZNF438*), histone methyltransferases (*ASH1L*, *MLL5*, *SETD5*, *SETMAR*) and acetyltransferase (*YEATS2*), other transcriptional regulators (*AKNA*, *BANP*, *CHD3*, *MEIS2*). Other interesting functions associated with these genes are: apoptosis (*BCL2L13*, *CASP8*, *C1orf201*), autophagy (*AMBRA1*, *ATG12*, *ATG13*, *ATG16L2*), splicing (*HNRNPH3*, *MBL2*), protein ubiquitination (*CNOT4*, *FBXW11*, *RFWD2*, *WDR20*) cytoskeleton and cytokinesis (*AURKA*, *EPS8L2*, *EPB41L2*, *LARP4*, *NPHP4*, *PLEC*, *TLN2*), primary cilium biogenesis [48] (*BBIP1*, *IFT140*, *KIAA0586*), intracellular trafficking/membrane trafficking (*ASAP1*, *KIF13A*, *MCOLN1*, *RAB17*, *TBC1D1*, *VPS29*), cell adhesion (*ARMC8*, *CD151*, *ELMOD3*, *LLP*, *VMP1*).

Taken together, these analyses suggest the strong effect of ERs binding on alternative splicing and confirming the key role of ERβ in BC cells.

## Discussion

In this study we investigated for the first time the effects of ERβ expression on estrogen-dependent pre-mRNA maturation and splicing. We used two different subclones of ERα-positive and estrogen-dependent MCF-7 cells stably expressing ERβ, and we found that expression of ERβ in these cells significantly affected the estradiol-dependent early transcriptional program and splicing pattern. In particular, introduction of ERβ caused loss of regulation of 25% of the estrogen-regulated genes. Moreover, a comparison between the ERβ + and the wt ERβ- cells showed that expression of ERβ caused loss of ERα-induced promoter switching in 75% of the genes, and of ERα-induced splicing in 72% of the genes.

Besides affecting ERα-dependent transcription and splicing, expression of ERβ caused the appearance of ERβ-specific estrogen-responsive transcription (550 genes) [Additional file 2: Tables S1-D], promoter-switching (61 genes) and differential splicing (28 genes) events, counting only the events that were present in both ERβ + lines. The biological functions of the ERβ-specific genes included DNA replication and repair, cell cycle, apoptosis and autophagy, DNA transcription, lipid metabolism, membrane metabolism, intracellular trafficking, mRNA maturation and translation, protein ubiquitination and sumoylation, cell signaling, confirming that a wide variety of cellular processes are affected by ERβ.

Based on our data, there are at least three different mechanisms by which ERβ can affect ERα-dependent transcription: (1) competition with ERα for binding to target gene promoters, in the form of competitive binding or heterodimerization, which can alter the recruitment of coregulators: the comparison between Chip-seq and RNA-seq data showed that the majority of the primary targets identified in this experiment were directly targeted by both ERα and ERβ (Figure S1), as expected also from data in the literature [49]; (2) gain of new binding sites that are not bound by ERα alone: a subset of binding sites appeared in the ERβ + cells but were not present in *wt* cells, again consistently with a previous report comparing ERα and ERβ genomic binding patterns in MCF-7 cells [49]; (3) secondary effects: expression of ERβ induced transcription and splicing of transcriptional regulators (most interestingly, the corepressor *NCOR2*, involved in gene repression by tamoxifen-bound estrogen receptor and by unliganded NRs such as the retinoic acid receptors) and of splicing factors, which can in turn affect estrogen-induced transcription and pre-mRNA maturation. For instance, ERβ induced promoter switching in the *PPM1G* gene, encoding a protein phosphatase responsible for dephosphorylation of pre-mRNA splicing factors [50]; the ERβ-specific alternate protein isoform from this gene (ENST00000350803) has an additional 17 amino acids in the N-terminal catalytic domain compared to the main isoform (ENST00000544412), which are likely to modulate the enzymatic function.

Even if the sole detection of splicing events does not give information on the biological consequences of ERβ effects on RNA splicing, it is tempting to speculate on the possible implications of ERβ-dependent splicing events on BC cells. Changes in splicing patterns can affect biological processes by many different mechanisms, including gain-of-function or functional switches, altered cellular localization, dominant negative effect, changes in protein/mRNA stability. For instance, expression of ERβ induced a promoter switch in the *USP7* gene, resulting in a shorter transcript (ENST00000535863) compared to the major isoform expressed in *wt* cells (ENST00000381886). This ERβ-specific isoform is missing the N-terminal 84 amino acids, a region of the protein of critical importance for interaction with substrates. This suggests the possibility of a switch in substrate affinity induced by ERβ. This is particularly interesting as USP7 is a deubiquitinating enzyme, responsible for removing ubiquitin chains from both the tumor suppressor p53 and its negative regulator Mdm2 [51], which instead is an ubiquitin ligase inducing degradation of p53. As USP7 binds both p53 and Mdm2 with the same N-terminal domain [51], the overall effect of its enzymatic activity is highly dependent on the relative affinity for the two targets. The ERβ-induced switch may alter this equilibrium, thus modulating such a relevant aspect of cancer biology as p53 stability. In the case of IFNγ Receptor 2, ERα induced expression of the full length transcript (ENST00000381995), while ERβ favored a switch toward truncated forms (ENST00000545369, ENST00000405436) lacking the transmembrane domain, and therefore predicted to be secreted as soluble forms in the extracellular environment, with the potential of acting as dominant negative modulators of interferon signaling. In another example (the gene *PSEN1*, involved in intramembrane proteolysis and cleavage of the intracellular domain of transmembrane proteins such as amyloid precursor protein and Notch and therefore a potential therapeutic target in BC [52]), the ERβ-induced splicing switch did not alter the open reading frame but caused the expression of a longer mRNA (ENST00000366783) compared to the isoform that was favored by ERα (ENST00000422240), possibly affecting the rate of translation or stability of the mRNA.

Another way of finding hints to the biological consequences of ERβ-specific splicing events described here is the reconstruction of pathways whose genes are differentially spliced following ERβ introduction in the cells. For instance, we found many genes involved in the Notch signaling pathway differentially spliced by ERβ: the above-mentioned PSEN2 is the catalytic subunit of the γ-secretase complex, responsible for intramembrane proteolysis of transmembrane receptors including Notch, resulting in the release of Notch Intracellular Domain (NICD), which migrates to the nucleus and regulates transcription of target genes [53]. Also, NCOR2 is bound to the unliganded CBF-1 transcription factor, a primary effector of Notch signaling which acts as a repressor in unstimulated cells, but is converted to an activator (dismissing the corepressor NCOR2) after binding the NICD [54]. Furthermore, POGLUT1 has recently been shown to be an endoplasmic reticulum O-glycosyl-transferase responsible for glycosylation of Notch and required for its function [55], and SLC35C2 is an endoplasmic reticulum transporter responsible for accumulation of GDP-fucose, which is used for Notch fucosylation, required for full activation [56]. Finally, SGK1 has recently been shown to be a negative regulator of Notch signaling by inhibiting γ-secretase activity and promoting Notch degradation [57], and MAGI1 has been shown to recruit Notch ligand Dll1 to cadherin-based adherens junctions, stabilizing it on the cell surface [58]. It is worth noting that the predominant Notch receptor expressed in these cell lines was Notch2, which was induced by E2 in *wt* cells (to a level slightly below the chosen cut-off threshold, but highly statistically significant: FPKM without E2: 49.826; FPKM with E2: 61.583, FC 1.236, q-value 0.0005), while its basal expression was lowered and its up-regulation smoothened in the ERβ + cells.

A possible modulation of the Notch pathway by ERβ is especially interesting as Notch is a known regulator of breast development and maintenance of breast stem cells [59]; alterations in the Notch pathway have been involved in breast carcinogenesis, and in particular the Notch pathway has been implicated in the development of triple negative BC (TNBC), a particularly aggressive form of BC which does not express ERα, progesterone receptor (PR) or HER2, and which has shown resistance to all known therapies [60]. Targeting Notch signaling has been proposed in TNBC. As these cancers are ERα-negative, hormonal treatment is not currently used for these patients; however, ERβ could be expressed in up to 50% of TNBCs [10,18], and its expression in TNBC has been associated with better prognosis [18]. Therefore, ERβ may represent a potential new therapeutic target in TNBC. The interrelation between ERβ and Notch in the development and prognosis of triple-negative BC should be investigated further in future research.

## Conclusions

In conclusion, whole-genome analysis of early transcription evens and mRNA processing associated with ERβ confirmed a relevant role for this receptor in modulating ERα-dependent transcription and splicing, but also identified novel, ERβ-specific transcription and splicing events, confirming a wide range of actions of ERβ in the biology of BC.

The data reported here confirm the complexity of estrogen action in BC cells and provide a comprehensive description of the effects of ERα and by ERβ on early transcription and splicing in hormone-responsive BC cells. More importantly, they provide a starting point to identify the events of ERβ-dependent splicing which are most significant for cancer biology.

## Methods

### Human hormone-responsive BC cells

Stable cell clones expressing either C-TAP-ERβ or N-TAP-ERβ (ERβ+) generated as previously described [23], and wild type (wt) MCF7 (ERβ-) cells were used for this study. All cell lines were maintained, propagated, hormone-starved for 5 days and analyzed for estrogen signaling as described earlier [21,45].

### Illumina RNA sequencing library preparation

Total RNA was extracted from hormone-starved cell cultures (+Ethanol -E2) or after 2 hours of stimulation with 10^-8^ M 17β-estradiol (+E2), as described previously [21]. RNA concentration in each sample was determined with a NanoDrop-1000 spectrophotometer and the quality assessed with the Agilent 2100 Bioanalyzer and Agilent RNA 6000nano cartridges (Agilent Technologies). Indexed libraries were prepared from 1 μg/ea. of purified RNA with TruSeq Stranded total RNA Sample Prep Kit (Illumina) according to the manufacturer’s instructions. Libraries were sequenced (paired-end, 2×100 cycles) at a concentration of 8 pmol/L per lane on HiSeq1500 platform (Illumina) with a coverage of >70 million sequence reads/sample on average.

### Read alignment and transcript assembly

TopHat v.2.0.10 [61] was used to align all reads including junction-spanning reads back to the human genome (Homo sapiens Ensembl GRCh37, hg19). To identify the differentially expressed and spliced genes and isoforms between ERβ- and ERβ + cell lines we used Cuffdiff v2.1.1 [44]. The parameters to define genes and isoforms as differentially expressed were the following: expression level threshold of 0.5 FPKM; *q*-value (FDR-adjusted *p*-value of the test statistic) ≤ 0.05 and |FC| ≥ 1.3. Moreover, to detect the ERβ- and ERβ + BC-specific splice events such as exon skip, exon inclusion, alternative splice sites and intron retention, we performed a direct comparison analysis using MATS v3.0.8 [37]. To filter events with at least 10% change in exon inclusion level we set the MATS cutoff *c*, representing the extent of splicing change one wishes to identify, to 0.1, and FDR ≤ 0.05 to filter the identified splice events.

### Data access

RNA-Seq data have been deposited in the Gene Expression Omnibus genomics data public repository (http://www.ncbi.nlm.nih.gov/geo/) with Accession Number GSE64590.

## Additional files

Additional file 1: Figure S1. Description: KEGG pathway (Kanehisa *et al.* Nucleic Acids Res 2014, 42:D199) analysis of ERα and ERβ interactors involved in Spliceosome Pathway. Blue and red boxes highlight ERα and ERβ interacting proteins, respectively. Additional file 2: Tables S1. A: Ct-ERβ gene list. Genes with a FPKM value ≥0.5, *q*-value ≤0.05 and |FC| ≥ 1.3 in the Ct-ERβ BC cell line. B: Nt-ERβ gene list. Genes with a FPKM value ≥0.5, *q*-value ≤0.05 and |FC| ≥1.3 in the Nt-ERβ BC cell line. C: MCF-7 wt gene list. Genes with a FPKM value ≥0.5, *q*-value ≤0.05 and |FC| ≥1.3 in the wt MCF-7 BC cell line. D: Commonly regulated genes in both ERβ + BC cell lines. Genes consistently regulated in both ERβ + BC cells with a *q*-value ≤0.05 and |FC| ≥1.3. E: Ct-ERβ isoform list. Isoforms with a FPKM value ≥0.5, *q*-value ≤0.05 and |FC| ≥1.3 in the Ct-ERβ BC cell line. F: Nt-ERβ isoform list. Isoforms with a FPKM value ≥0.5, *q*-value ≤0.05 and |FC| ≥1.3 in the Nt-ERβ BC cell line. G: MCF-7 wt isoform list. Isoforms with a FPKM value ≥0.5, FDR ≤0.05 and |FC| ≥1.3 in the wt MCF-7 BC cell line. Additional file 3: Tables S2. A: List of exon skipping events in Ct-ERβ, B: List of mutually exclusive exons in Ct-ERβ, C: List of retained introns in Ct-ERβ, D: List of alternative 3’ splice sites in Ct-ERβ, E: List of alternative 5’ splice sites in Ct-ERβ, F: List of exon skipping events in Nt-ERβ, G: List of mutually exclusive exons in Nt-ERβ, H: List of retained introns in Nt-ERβ, I: List of alternative 3’ splice sites in Nt-ERβ, J: List of alternative 5’ splice sites in Nt-ERβ, K: List of exon skipping events in wt MCF-7, L. List of mutually exclusive exons in wt MCF-7, M: List of retained introns in wt MCF-7, N. List of alternative 3’ splice sites in wt MCF-7, O: List of alternative 5’ splice sites in wt MCF-7. Additional file 4: Table S3. Discordant Isoforms ERβ+/ERβ-. List of all isoforms with opposite regulation pattern in ERβ+/ERβ-. Additional file 5: Tables S4. A: Differential Promoter Usage Ct-ERβ. Gene list with differential promoter usage (DPU) in the Ct-ERβ BC cell line (FDR ≤0.05). B: Differential Promoter Usage Nt-ERβ. Gene list with DPU in the Nt-ERβ BC cell line (FDR ≤0.05). C: Differential Promoter Usage MCF-7 wt. Gene list with DPU in the wt MCF-7 BC cell line (FDR ≤0.05). D: Differential Spliced Gene Ct-ERβ. Gene list differentially spliced (DSGs) in the Ct-ERβ BC cell line (FDR ≤0.05). E: Differential Spliced Gene Nt-ERβ. Gene list differentially spliced (DSGs) in the Nt-ERβ BC cell line (FDR ≤0.05). F: Differential Spliced Gene MCF-7 wt. Gene list differentially spliced (DSGs) in the wt MCF-7 BC cell line (FDR ≤0.05). Additional file 6: Table S5.
*PSD3* TSSs expression values. Transcript starting sites (TSSs) expression value list of the *PSD3* gene, expressed in FPKM with relative FC, FDR and *p*-value in Ct-ERβ, Nt-ERβ and MCF-7 wt BC cell lines. Additional file 7: Tables S6. A: ERα binding sites in Ct-ERβ. Gene list of DSGs including ERα binding sites in a 10 kb window around the gene in the Ct-ERβ BC cell line. B: ERβ binding sites in Ct-ERβ. Gene list of DSGs including ERβ binding sites in a 10 kb window around the gene in the Ct-ERβ BC cell line. C: ERα binding sites in Nt-ERβ. Gene list of DSGs including ERα binding sites in a 10 kb window around the gene in the Nt-ERβ BC cell line. D: ERβ binding sites in Nt-ERβ. Gene list of DSGs including ERβ binding sites in a 10 kb window around the gene in the Nt-ERβ BC cell line. E: ERα binding sites in MCF-7 wt. Gene list of DSGs including ERα binding sites in a 10 kb window around the gene in the wt MCF-7 BC cell line. Additional file 8: Figure S2. Heat map representing the clustered density matrix of ERα and ERβ binding sites. Chromatin immunoprecipitation of ERα and ERβ in Ct-ERβ and *wt* MCF-7 cells show different ER binding profiles (highlighted by square brackets). In the clustering, each line represents a genomic location of a binding site with its surrounding ±1.5 kb region. This matrix was subjected to *k*-means clustering.

## Abbreviations

ER: Estrogen receptor
Nt-ERβ: N-TAP-estrogen receptor beta
Ct-ERβ: C-TAP-estrogen receptor beta
E2: 17β-estradiol
BC: Breast cancer
FPKM: Fragment per kilobases of exon per million
DSGs: Differentially spliced genes
TSSs: Transcripts starting sites
DPU: Differential promoter usage
