# Supplementary figures and images for: Estrogen receptor beta impacts hormone-induced alternative mRNA splicing in breast cancer cells
Source: BMC Genomics. 2015 May 9;16(1):367. doi: 10.1186/s12864-015-1541-1 (PMC4424892; doi:10.1186/s12864-015-1541-1)

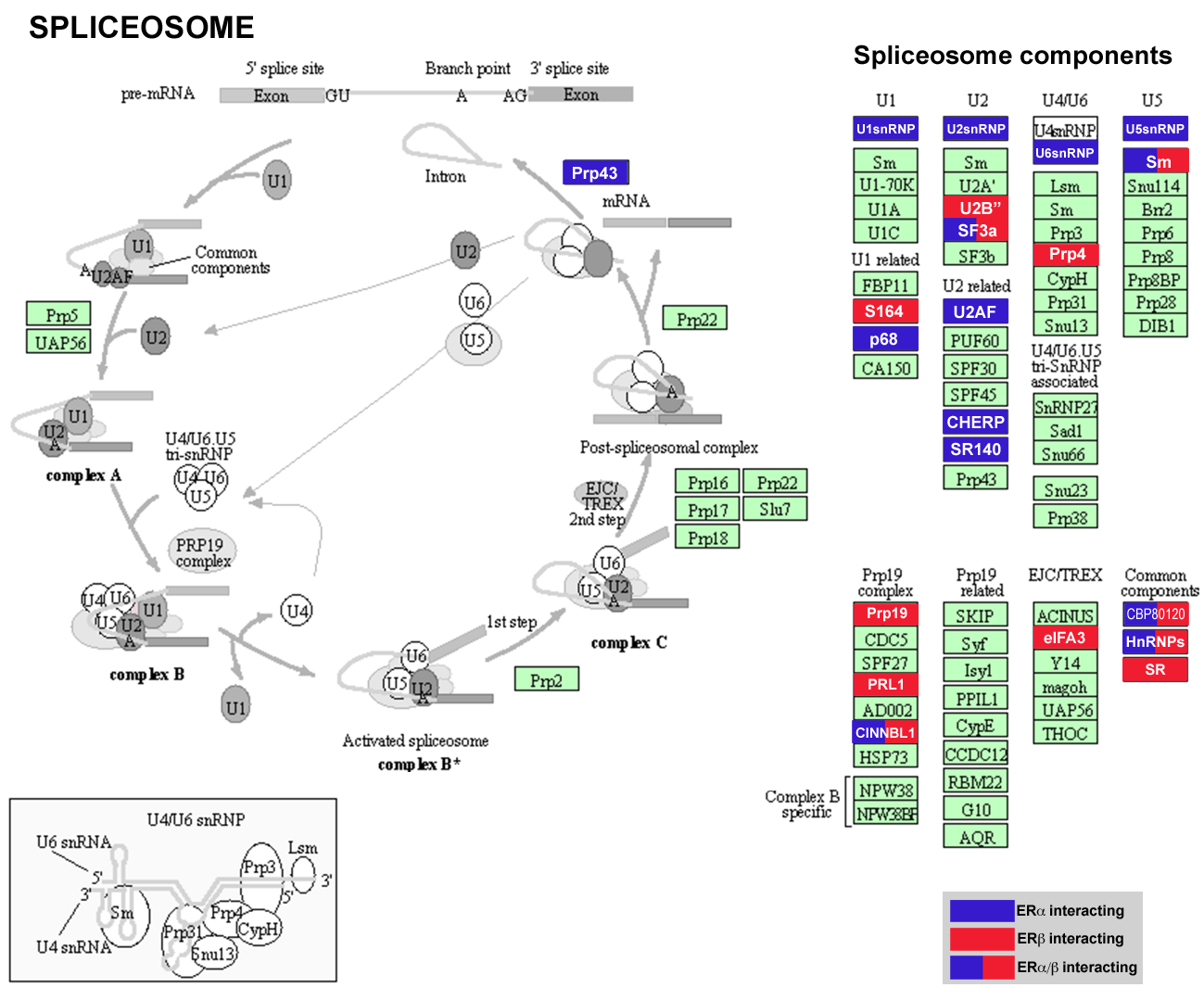

Supplement: Additional file 1: Figure S1. — Description: KEGG pathway (Kanehisa et al. Nucleic Acids Res 2014, 42:D199) analysis of ERα and ERβ interactors involved in Spliceosome Pathway. Blue and red boxes highlight ERα and ERβ interacting proteins, respectively. [file 12864_2015_1541_MOESM1_ESM.tiff]

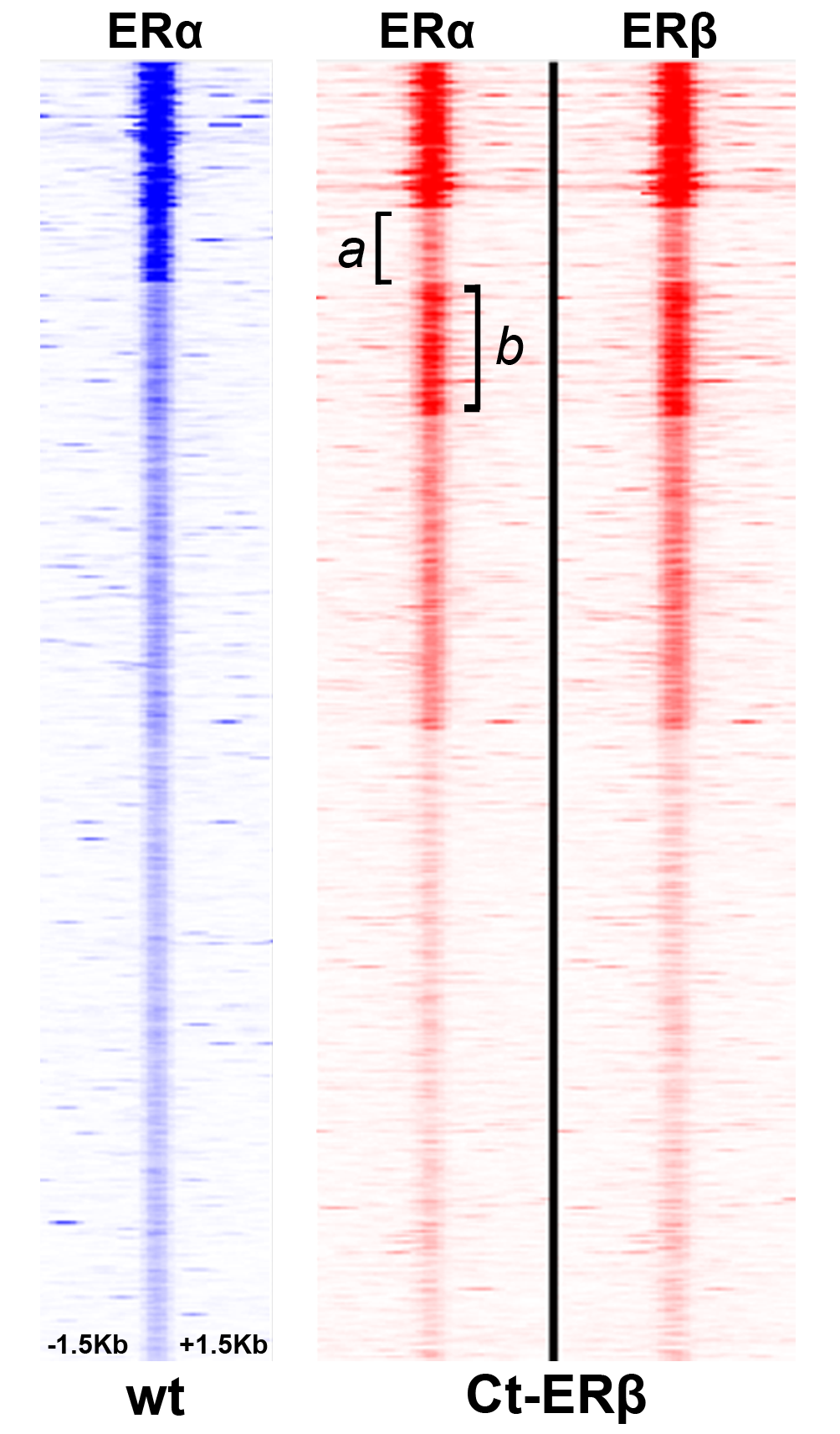

Supplement: Additional file 8: Figure S2. — Heat map representing the clustered density matrix of ERα and ERβ binding sites. Chromatin immunoprecipitation of ERα and ERβ in Ct-ERβ and wt MCF-7 cells show different ER binding profiles (highlighted by square brackets). In the clustering, each line represents a genomic location of a binding site with its surrounding ±1.5 kb region. This matrix was subjected to k-means clustering. [file 12864_2015_1541_MOESM8_ESM.tiff]
